# Explainable computational framework for subtype classification and biomarker identification in head and neck squamous cell carcinoma using machine learning

**DOI:** 10.1371/journal.pone.0360612

**Published:** 2026-11-04

**Authors:** Anagha Rajan, Christopher Leela Biji, Mangalam Goutam Sinha, Iyappan Ramalakshmi Oviya

**Affiliations:** 1 Department of Sciences, Amrita School of Engineering, Amrita Vishwa Vidyapeetham, Chennai, India; 2 School of Computer Science and Engineering, Vellore Institute of Technology, Vellore, Tamil Nadu, India; 3 Department of Computer Science and Engineering, Amrita School of Computing, Amrita Vishwa Vidyapeetham, Chennai, India; Xiangya Hospital Central South University, CHINA

## Abstract

The precise classification of basal, classical, atypical, and mesenchymal subtypes of Head and Neck Squamous Cell Carcinoma (HNSCC) is crucial for advancing precision oncology. Our research aims to develop an explainable subtype classification model for HNSCC.These molecular subtypes have been previously defined; reliable and meaningful computational methods for the classification of molecular subtypes and prioritization of molecular biomarkers for each subtype do not exist. We developed an explainable artificial intelligence (XAI) model that classifies molecular subtypes based on combined transcriptomic profiles from The Cancer Genome Atlas (TCGA-HNSC) and Gene Expression Omnibus (GSE65858). SHapley Additive exPlanations (SHAP) were used to quantify the importance of genes for model predictions. Subsequently, genes with high predictive importance were analyzed in protein-protein interactions using STRING, and hub genes were identified by Maximum Clique Centrality (MCC) and used for the identification of candidate biomarkers for each of the subtypes. We employed machine learning methods such as Random Forest (RF), Logistic Regression (LR), Decision Tree (DT), AdaBoost (AB), Support Vector Machine (SVM), K-Nearest Neighbours (KNN), Gaussian Naive Bayes (GNB), XGBoost (XGB), and Gradient Boosting (GB). The XGBoost model achieved an accuracy of 95% after hyperparameter tuning and cross-validation. Biologically relevant subtype-specific biomarkers associated with each molecular subtype were identified. To highlight the potential biological functions of these biomarkers, pathway analysis was performed. Further assessment of these genes, such as survival analysis and druggability, showed promise for the development of targeted therapies.

## Introduction

Head and Neck Squamous Cell Carcinoma (HNSCC) exhibits diverse molecular and clinical characteristics [1,2]. The difficulty in early diagnosis of the disease has created an urgent need for the development of targeted therapies and also enhanced the survival rates of patients [3,4]. Recent breakthroughs in high-throughput sequencing, along with the application of machine learning (ML) and deep learning (DL) algorithms, have changed the landscape of cancer research by providing detailed molecular signatures of the disease [5]. The implementation of recent technologies has helped identify the gene expression patterns associated with various cancer types, paving the way for the subclassification of HNSCC into specific molecular subtypes. Each molecular subtype exhibits a different set of biological and clinical traits that affect the response to treatment, metastasis potential, and prognosis [6]. Extensive gene expression analyzes, often based on The Cancer Genome Atlas (TCGA) data, indicate that HNSCC tumors are classified into four transcriptomic subtypes: atypical, classical, basal, and mesenchymal. These subtypes have distinct genomic alterations, tumor microenvironment features, and clinical phenotypes, validated in several independent patient cohorts, such as radiomic studies and single-cell transcriptomic profiling [7]. The basal subtype is related to epithelial basal cells and adhesion-related activities and partially undergoes epithelial-mesenchymal transition (EMT). Additionally, the mesenchymal subtype is upregulated in EMT, stromal, and immune-stromal signatures, which are typically associated with therapeutic resistance and worse prognoses in a range of squamous and gastrointestinal tumors. The classical subtype is characterized by a high proportion of mutations in TP53 in combination with the dysregulation of pathways related to oxidative stress and xenobiotic metabolism. Atypical tumors are displayed in immune-related and differentiation pathways and have a greater prevalence of Human Papillomavirus (HPV) positive oropharyngeal disease [8]. These subtypes are classified based on the activity of the respective genes in specific cancer-linked processes. Molecular subtyping in clinical settings still faces several challenges. One of the major issues is the large amount of gene expression data that must be analyzed. Microarrays and RNA sequencing are among the techniques that involve the processing of numerous genes, but only a few patient samples are available for analysis. ML methods are used to address this issue. ML algorithms can handle large-scale and high-dimensional datasets and extract meaningful features from samples [9,10].

## Literature survey

The implementation of ML and DL approaches in oncology, especially in the molecular subtype classification of HNSCC, has made significant progress. Various studies have integrated multi-omics data, such as genomics, proteomics, metabolomics, and transcriptomics, with efficient ML algorithms, such as random forests, support vector machines, elastic net, and deep neural networks. The researchers discovered two stable molecular subtypes, CS1 and CS2, across four large HNSCC cohorts, by applying consensus clustering in combination with an elastic net-driven 30-gene prognostic signature prediction. The ML models linked these subtypes with different patterns of immune infiltration and varied chemotherapy and immunotherapy responses, thus demonstrating the biological relevance and precision oncology potential of subtype classifications [11]. The ensemble classifier, which was composed of five machine learning models, achieved 100% accuracy in subtyping HPV-positive HNSCC tumors into immune-strong (IMU) and highly keratinized (KRT) molecular subtypes on a test set of 229 RNA-seq samples. The IMU subtype is characterized by the presence of immune activation and epithelial-mesenchymal transition (EMT), which has lower resistance to radiation and better clinical outcomes [12]. The use of DL and ML techniques on single-cell RNA-sequencing data in the classification of tumor vs. normal showed the highest accuracy; in addition, the model allowed the discovery of biomarker genes specific to each cell type [13]. A deep learning framework combining different modalities was built using vision transformers that had been pretrained with masked autoencoders (MAE) and attention modules and was able to combine CT imaging with clinical variables to classify AJCC (American Joint Committee on Cancer) stages in HNSCC. The model achieved an accuracy of up to 80.4% and 0.81 AUC on a balanced dataset, respectively, and also proves the capability of adapting to data imbalance by using tailored attention mechanisms [14]. The diverse field of AI applications in diagnosis, prognosis, radiomics, and treatment decision-making in HNSCC, thus predicting the role of integrated data-driven models in future clinical pathways [15].. In addition, scientists have used multi-omics datasets to uncover four molecular HNSCC subtypes (classical, mesenchymal, basal, and atypical), each with its characteristic immune and epithelial signaling features, thus providing biological insights and the identification of vulnerabilities specific to each subtype [16]. Using single-cell RNA sequencing data, machine learning models such as XGBoost, Random Forest, and deep learning predicted the HPV status with an area under the receiver operating characteristic (AUROC) of 0.91 and 0.83, respectively [17]. Most models depend on bulk transcriptomic datasets, which hide the intratumoral heterogeneity that is important in the pathology of HNSCC. Typically, datasets are limited in size, resulting in imbalanced classes. This imbalance often causes machine learning classifiers to overfit, thereby diminishing their ability to generalize [18]. Furthermore, combining genetic, epigenetic, transcriptomic, proteomic, and clinical data is a major task in developing fully integrated and molecular subtyping frameworks. Classifying molecular subtypes in HNSCC through the implementation of machine learning techniques helps predict treatment response and can guide us in the era of precision oncology [19]. This literature survey brings together critical studies, sources of data, ML/DL methods, predictions of subtypes, validation techniques, performance measures, biological analyzes, and future directions, providing a strong basis for subtype classification of molecular subtypes in head and neck cancer.

This study aimed to create a machine learning model capable of predicting the four molecular subtype classifications of HNSCC, utilizing machine models and interpreting the results with SHAP. This framework allows for the recognition of subtype-specific gene signatures and comprehensive bioinformatics analysis of the highlighted biomarkers.

The main objectives of the present study are as follows:

(i) Compilation of cross-platform transcriptomic datasets from TCGA-HNSC and GEO for the HNSCC molecular subtyping (TCGA-HNSC + GEO).
(ii) An explainable artificial intelligence (XAI) framework was proposed for the classification of the molecular subtypes of head and neck carcinoma: atypical, basal, classical, and mesenchymal.
(iii) XAI-based feature selection for subtype-specific biomarker discovery.
(iv) Pathway analysis on the selected biomarkers revealed the biological relevance of the biomarkers.
(v) The druggability and resistance of the discovered biomarkers, along with their role in predicting survival rate.

By understanding the specific characteristics of each subtype, researchers and clinicians can develop novel and personalized treatment strategies, potentially improving patient outcomes and overall survival rates.

## Methods

### Data Collection

HNSCC subtype data were downloaded from two publicly available repositories, TCGA (https://www.cancer.gov/ccg/research/ genome-sequencing/tcga) and GEO, on September 20, 2024 [20]. The vital goal of data acquisition is to ensure a high-quality dataset of gene expression samples from different HNSCC molecular subtypes. The number of samples selected was based on the completeness of the gene expression profile and the availability of subtype annotations. The study was based on publicly available HNSCC gene expression datasets: TCGA-HNSC RNA-seq and GSE65858 microarray. After quality assessment and sample selection, the TCGA-HNSC cohort consisted of 322 samples, and the GSE65858 dataset consisted of 270 samples. Samples were classified into atypical, basal, classical, and mesenchymal molecular subtypes with the included metadata. The total sample count used in this study is presented in the Table 1. The use of both datasets provides a better and more reliable group for machine learning in downstream analyzes. The data were pre-processed to filter the subtype samples for further analysis. The overall workflow of this study is illustrated in Fig 1.

**Table 1:** Distribution of samples across molecular subtypes and datasets.

Dataset	Total Samples	Atypical	Basal	Classical	Mesenchymal
TCGA-HNSC	322	68	87	49	74
GEO	270	73	84	30	83
Combined (TCGA-HNSC + GEO)	592	141	171	79	157

**Fig 1:**
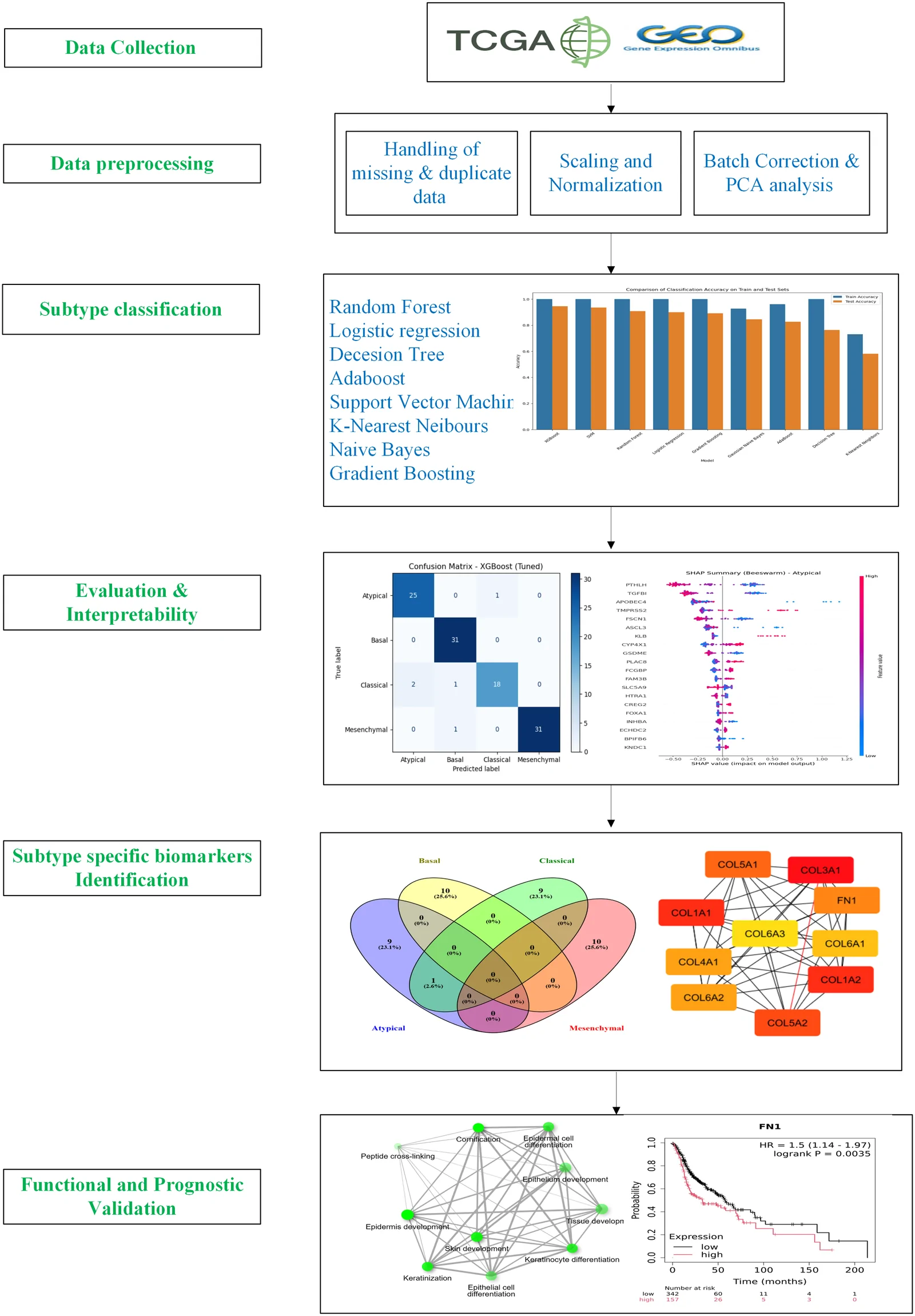
Overall workflow of the proposed subtype classification approach for HNSCC.

### Data pre-processing

Data processing is a vital process that guarantees data integrity and reliability. RNA sequencing (RNA-seq) data collected from the TCGA-HNSC cohort and microarray expression data from the GSE65858 cohort were processed using a standard pipeline. The expression data for TCGA-HNSC were accessed as raw HTSeq-count matrices via the TCGAbiolinks package, whereas the expression data for GSE65858 were imported as a processed gene expression matrix. The gene identifiers from each dataset were mapped to their official HGNC gene symbols (using gene annotation information), and only genes common to both datasets were used for subsequent analyzes. Lowly expressed genes were filtered from the TCGA RNA-seq dataset using the edgeR package, with a cutoff of counts-per-million (CPM)> 1 in at least 2 samples. The filtered count matrix was normalized using the Trimmed Mean of M-values (TMM) method implemented in calcNormFactors() and subsequently transformed to voom using the limma package to model the mean-variance relationship of RNA-seq count data and to produce precision weights for linear modelling in downstream analysis. Downstream analysis was conducted with only common genes between the two datasets (1673). Common genes were subsequently retained after preprocessing and filtering for downstream analysis of differential expression, machine learning, explainable artificial intelligence (XAI) and network analysis [21,22].

The filtered count matrix was normalized using the Trimmed Mean of M-values (TMM) method implemented in calcNormFactors() and subsequently transformed to voom using the limma package to model the mean-variance relationship of RNA-seq count data and to produce precision weights for linear modelling in downstream analysis. Batch effects due to differences between the RNA-seq and microarray platforms were removed by applying the ComBat function to the sva package. The sequencing platform (TCGA or GEO) was indicated as the batch variable, and molecular subtype was included as a biological covariate in the model matrix to retain the biological variation within each molecular subtype in the empirical Bayes batch correction (prior.plots = FALSE, par.prior = TRUE). Principal component analysis (PCA) was performed before and after ComBat correction to assess the effectiveness of the batch effect correction. Before correction, the samples were mainly grouped by their experimental platform, suggesting the presence of platform-associated batch effects. After the removal of technical variation (ComBat correction), the differences between the TCGA and GEO samples were significantly reduced, with cluster grouping still occurring by molecular subtypes, indicating successful correction of the technical variation without masking expression patterns. For model development, normal samples were excluded, and the gene expression matrix was split into 80% training and 20% testing groups using the train_test_split function (random_state = 42) [23,24].

To reduce the class imbalance problem, SMOTE was used only for the training set to create balanced classes for each subtype without affecting the independence of the test set [25,26]. To overcome the issue of class imbalance, the Synthetic Minority Oversampling Technique (SMOTE) was applied. After stratified partitioning of the integrated dataset into 80% training and 20% test sets, the SMOTE was only applied to the training set to create minority synthetic samples of the molecular subtypes, with the independent test set not modified. This method eliminated the information leakage problem in synthetic samples in the test set and provided an unbiased test of the model performance. The balanced training dataset was then used to develop the model, and the performance was evaluated on the original unseen test dataset.

### Model selection

For the molecular head and neck subtype classification, nine different machine learning algorithms, including Random Forest (RF), Logistic Regression (LR), Decision Tree (DT), AdaBoost (AB), Support Vector Machine (SVM), K-Nearest Neighbours (KNN), Gaussian Naive Bayes (GNB), XGBoost (XGB), and Gradient Boosting (GB) were applied [27]. LR, a linear classifier, was employed to develop baseline relationships between gene expression features and HNSCC subtypes. The SVM is used with a nonlinear kernel to extract high-dimensional and complex decision boundaries. DT and RF models were used as non-linear and tree-based models to learn hierarchical feature interactions. AB and GB ensemble boosting algorithms have been introduced to improve the accuracy of predictions, correct errors of classification, and focus on capturing the most informative biomarkers and distance-based predictors, such as KNN. KNN has been introduced to measure the local similarity pattern in the feature space, and GNB, which is a probabilistic model, has been used as a simple distance-based predictor under conditions of the independence assumption. This set of classifiers collectively made it possible to compare a strong linear and nonlinear decision function and ensemble approach to identify HNSCC subtypes. Each model was trained using samples from a predefined and consistent dataset. A cross-validation process was performed to ensure the robustness of these results. The performance metrics used for the overall performance computation included accuracy, precision, recall, and F1 scores. The best classification model was selected for further evaluation.

### Model Explainability and Interpretation

We utilized the SHAP model to ensure the transparency and interpretability of HNSCC subtype classification. This method supports the detection of pivotal genes that contribute to the subtyping process and reveals the biological basis for categorization [28]. SHapley Additive exPlanations (SHAP) is a game-theoretic method that describes the individual model predictions by computing the marginal contribution of the individual features to the prediction (i.e., the Shapley value), thus offering both local and global explanations [29].

The mean absolute SHAP value for gene j was calculated as shown below.


$$\begin{array}{c} M_{j}\text{\textasciitilde{}}=\text{\textasciitilde{}}\frac{1}{N}\text{\textasciitilde{}}\sum_{i-1}^{N}\text{\textasciitilde{}}\left|\emptyset_{ij}\right| \end{array}$$ (1)

Here, ‘Mj’ represents the global importance score, N is the total number of samples, ϕij is the SHAP value of gene j for sample i.

The absolute SHAP values were taken into consideration, as positive and negative values indicate different directions of influence, and their absolute size signifies the predictive power of the feature.

Subsequently, genes were ranked according to


$$\begin{array}{c} M(1)\;\geq \;M(2)\;\geq \;\cdots \;\geq \;M(p) \end{array}$$

where p denotes the total number of genes.

### Percentile-Based Feature Selection

To increase the statistical robustness and reproducibility of the results, a percentile-based feature selection approach was used. Each threshold percentile was independently assessed using atypical, basal, classical, and mesenchymal models. The calculated thresholds were 0.00838, 0.01063, 0.00830, and 0.01149, respectively. These values varied only slightly between the subtypes; therefore, a common cut-off value was chosen for consistency and reproducibility throughout the study: 0.01. Thus, only genes with a score of ≥ 0.01 (Mj ≥ 0.01) were retained. The percentile-based approach is designed to exclude features that do not substantially contribute to the model’s predictions and preferentially include the top 5% of features, that is, the genes with the highest SHAP values [30].

### Identification of significant genes in the subtypes

Subsequently, after conducting SHAP analysis, we compiled a list of genes linked to each molecular subtype of HNSCC. The genes with SHAP values ≥ 0.01 were retained, and these were the only genes that had a major influence on the model’s predictions. The remaining genes were used to construct a protein–protein interaction (PPI) network using the STRING database. The network was constructed and analyzed using Cytoscape with the CytoHubba plugin. Hub genes were determined by applying the Maximum Clique Centrality (MCC) algorithm in the CytoHubba plugin of Cytoscape. MCC assigns higher scores to genes involved in multiple subnetworks with strong interconnections.

The ability of MCC to capture essential proteins has been shown to be better than that of traditional network centrality measures. MCC assigns a score to a node based on its membership in the largest cliques of nodes and hence identifies proteins that are integrated within large interconnected functional modules that are frequently linked to important biological functions. Hence, the MCC was chosen as one of the main hub ranking algorithms in the present study. The top 10 genes with the highest MCC scores were chosen as candidate genes, as they were the most highly interconnected proteins within the molecular subtype-specific interaction network [31]. Finally, the subtype-specific hub gene sets were compared using Venn analysis to obtain the biomarkers specific to each subtype and those shared among multiple molecular subtypes of HNSCC. These prioritized genes were then subjected to functional and biological interpretation and enrichment analyzes.

This combined method provides a strong basis for understanding the decision-making process of machine learning models and broadens our comprehension of subtype-specific molecular mechanisms.

### Pathway enrichment analysis using ShinyGO

The enrichment analysis was performed using the ShinyGO v0.81 web server to investigate the biological pathways associated with the prioritized subtype-specific genes. Official HGNC gene symbols were used to upload the gene lists, and Homo sapiens was selected as the reference organism. ShinyGO performed the Kyoto Encyclopedia of Genes and Genomes (KEGG) pathway over-representation analysis using the hypergeometric test. The P-values were corrected for multiple hypothesis testing using the false discovery rate (FDR) method according to Benjamini and Hochberg. Pathways with adjusted P values (FDR) < 0.05 were regarded as significantly enriched KEGG pathways. The significantly enriched pathways were then functionally annotated to determine biological mechanisms and therapeutic targets for each molecular HNSCC subtype [32].

### Druggability assessment of the biomarkers using DGID

The druggability assessment of the biomarker set of HNSCC was conducted by using the Drug–Gene Interaction Database, an openly available database https://www.dgidb.org (accessed in March 2025) [33] that integrates gene–drug interaction data extracted from a range of expert-curated resources, including Pharos, GuideToPharmacology, HingoraniCasas, HopkinsGroom, RussLampel, GO, and dGene, respectively. This approach allowed the discovery of HNSCC biomarkers of biological relevance with translational potential for therapeutic innovation and repurposing.

### Survival analysis using kmplotter

The survival significance of the prioritized genes was assessed in a head and neck squamous cell carcinoma (HNSCC) patient group using the Kaplan–Meier Plotter database with overall survival (OS) as the clinical endpoint [34]. The patients were divided into high- and low-expression subgroups based on the platform-provided auto-selected optimal expression cutoff. The molecular subtype of the tumor, stage, gender, race, histological grade, mutation burden, neoantigen load, and immune cell infiltration were not restricted; thus, all eligible cases of HNSCC available in the Kaplan–Meier Plotter database at default analysis conditions were used, such as age, sex, and race. The log-rank test was used to compare Kaplan–Meier survival curves, and the Cox proportional hazards model was used to estimate hazard ratios (HRs) and 95% confidence intervals (CIs) within the platform. A difference with a P value < 0.05 was regarded as statistically significant.

## Results

The PCA plots serve as a clear and vivid visual representation of the batch correction effect between the two datasets, TCGA-HNSC and the GEO dataset (GSE65858). The pre-processing step showed a sharp separation between the two datasets along the first principal component (PC1), with the TCGA samples grouped on one side and the GSE samples on the other. This distinct demarcation highlights the existence of a considerable batch effect, which implies that the major part of the PCA-captured variation was due to the differences in technical and experimental factors (such as sequencing platforms, sample preparation, and data normalization), rather than being an indicator of biological variation. Moreover, if the datasets are integrated in the uncorrected state, the readings would be biased because the clustering pattern reflects the dataset source rather than revealing the biologically relevant subtypes. Once batch correction was applied, the PCA plot showed a significant improvement in data integration (refer to S1 Fig).

The volcano plot describes the significant genes present in the HNSCC subtypes illustrated in Fig 2. Red dots are denoting the significant genes in the given samples and the grey dots are indicating the non significant genes. The atypical subtype showed a sharp division between upregulated and downregulated genes, reflecting major transcriptional dysregulation compared to normal tissues. The basal subtype has a greater number of significantly upregulated genes, indicating an active oncogenic signaling profile. In contrast, the mesenchymal and classical subtypes revealed an enormous number of genes related to epithelial-to-mesenchymal transition (EMT) and tumor invasiveness.

**Fig 2:**
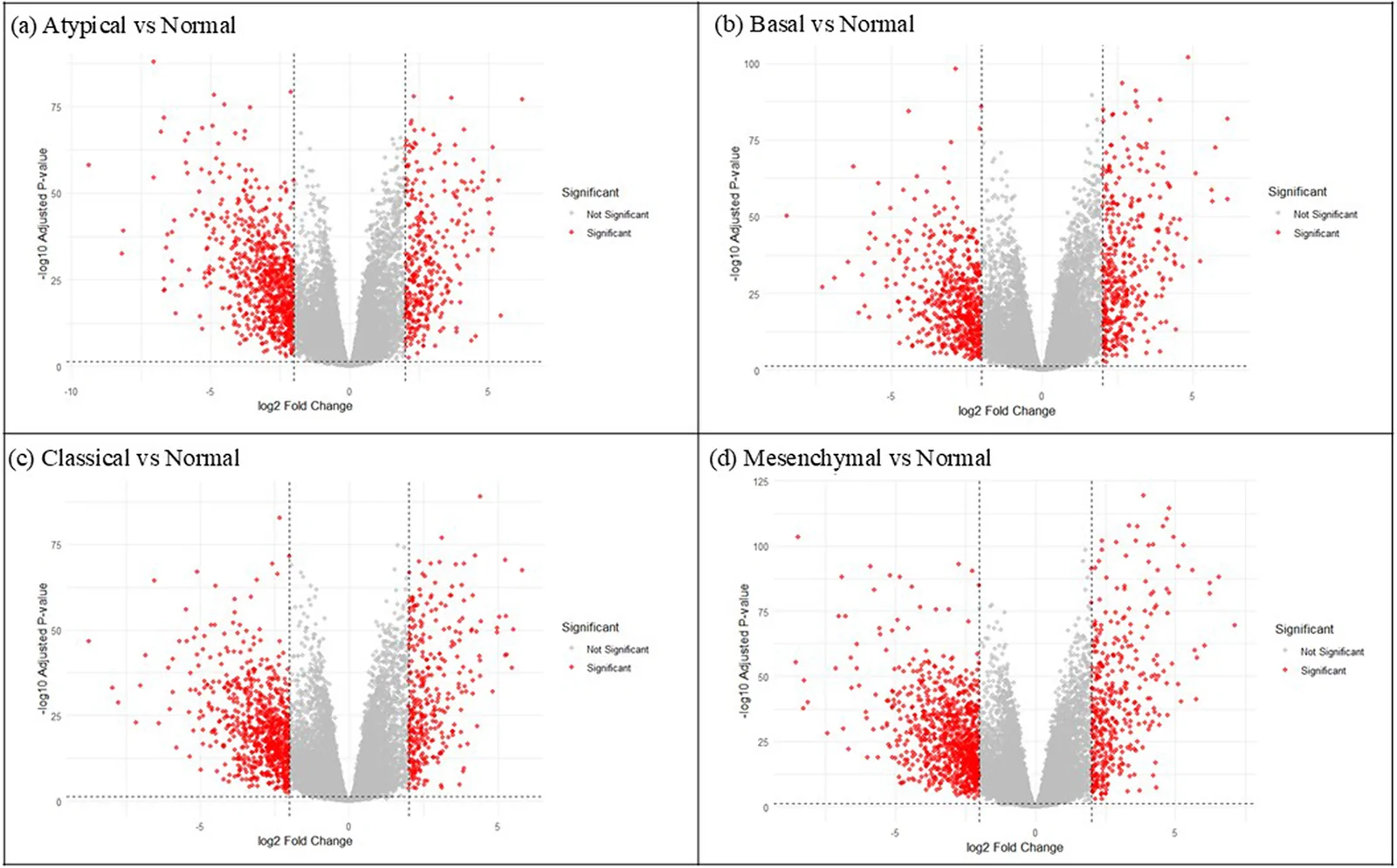
Volcano plot showing differential gene expression in each HNSCC subtype (a) Volcano plot for atypical vs normal samples (b) Volcano plot for basal vs normal samples (c) Volcano plot for classical vs normal samples. (d) Volcano plot for mesenchymal vs normal samples.

The evaluation metrics used, such as precision, accuracy, recall, and F1 score. The classification accuracy comparison of subtypes using nine machine learning models is shown in Table 2. These findings highlight the effectiveness of the models in capturing data complexity. The results show a remarkable improvement in the accuracy of molecular subtype classification of HNSCC. The training and testing accuracies of all the models are summarized in Fig 3.

**Table 2:** Comparative performance of nine machine learning algorithms for HNSCC molecular subtype classification.

ML	HNSC-Subtype	Precision (%)	Recall (%)	F1-score (%)
Techniques				
**LR**	Atypical	88	88	88
	Basal	88	97	92
	Classical	89	76	82
	Mesenchymal	94	94	94
**DT**	Atypical	86	69	77
	Basal	68	90	78
	Classical	81	62	70
	Mesenchymal	78	78	78
**RF**	Atypical	86	96	91
	Basal	84	100	91
	Classical	100	71	83
	Mesenchymal	100	91	95
**AdaBoost**	Atypical	95	81	88
	Basal	74	90	81
	Classical	83	71	77
	Mesenchymal	84	84	84
**Gradient Boosting**	Atypical	88	85	86
	Basal	88	97	92
	Classical	84	76	80
	Mesenchymal	94	94	94
**SVM**	Atypical	92	92	92
	Basal	89	100	94
	Classical	95	86	90
	Mesenchymal	100	94	97
**KNN**	Atypical	62	62	62
	Basal	54	61	58
	Classical	100	38	55
	Mesenchymal	51	66	58
**GNB**	Atypical	81	85	83
	Basal	78	90	84
	Classical	90	90	90
	Mesenchymal	92	75	83
**XGBoost**	Atypical	93	96	94
	Basal	94	100	97
	Classical	90	86	88
	Mesenchymal	100	94	97

**Fig 3:**
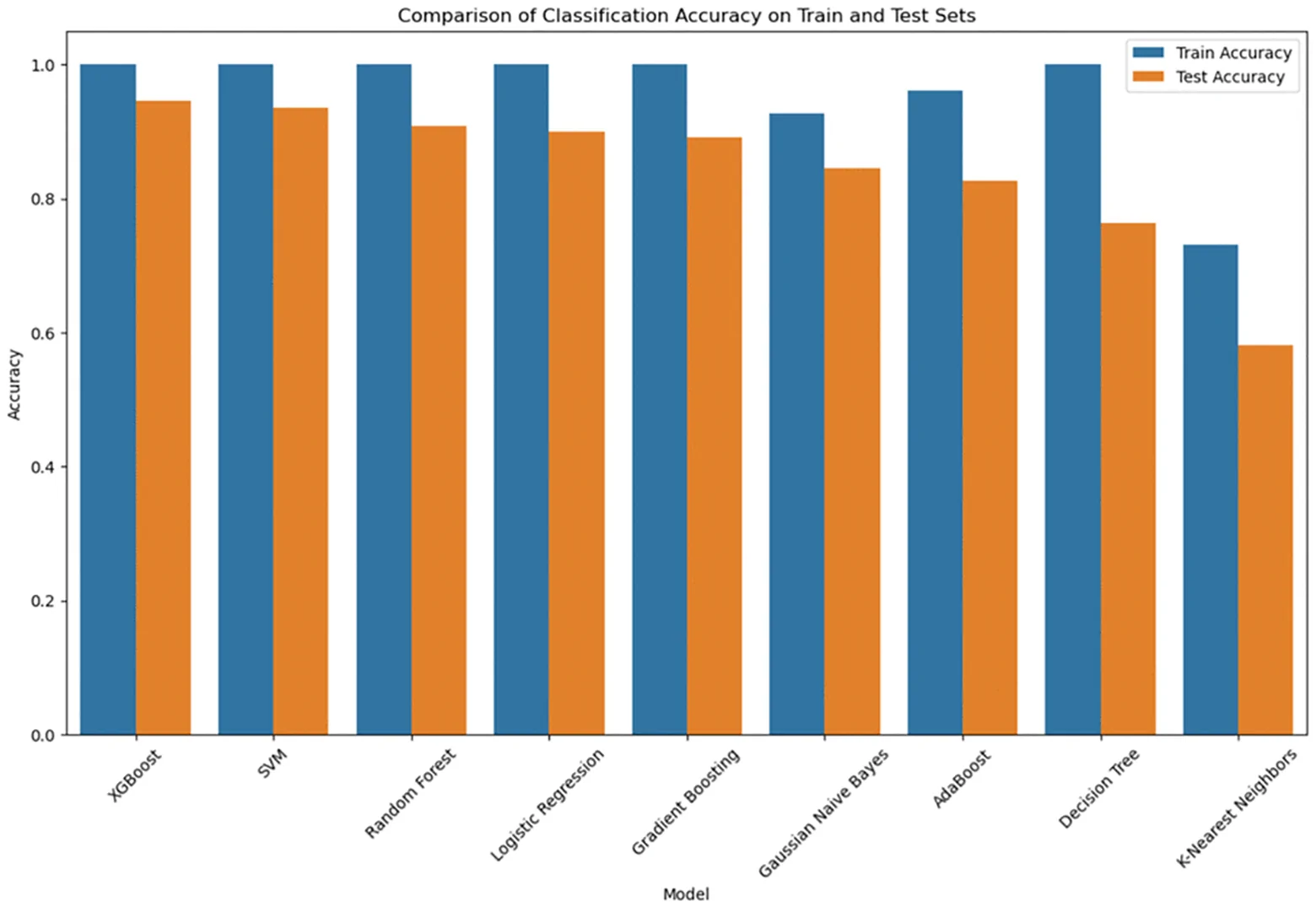
Comparison of accuracy on the train and test datasets for subtype classification.

From these ML models, XGBoost outperformed all other classifiers in terms of testing accuracy (94.55%), followed by SVM (93.64%) and Random Forest (90.91%). The confusion matrices of all the models are given in S2 Fig. Based on this result, XGBoost, SVM and RF were further tested using cross validation to assess their robustness and generalizability. For classification, XGBoost was found to be the most accurate model with a cross-validation accuracy of 95% and thus was used for all further analyzes; the details of evaluation parameters are given in Table 3. Therefore, subsequent explainable artificial intelligence (XAI) and biomarker identification were conducted using XGBoost. The confusion matrix and ROC curve of the XGBoost are given in Fig 4.

**Table 3:** Classification of XGBoost after the fine-tuning process.

Subtype	Precision (%)	Recall (%)	F1-score (%)
Atypical	93	96	94
Basal	94	100	97
Classical	95	86	90
Mesenchymal	100	97	98

**Fig 4:**
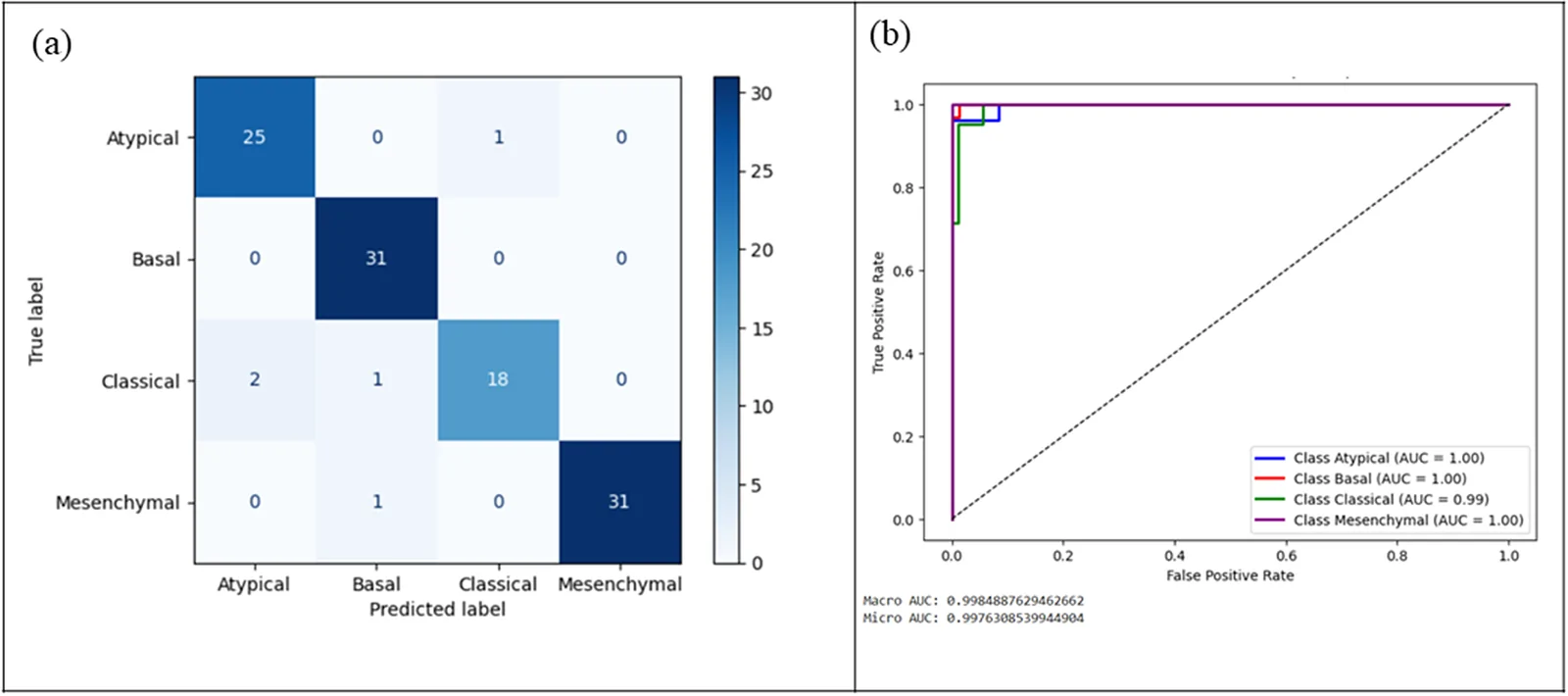
(a) Performance evaluation of the tuned XGBoost model for molecular subtype classification; (b) Receiver Operating Characteristic (ROC) curves of the tuned XGBoost classifier after cross-validation.

The SHAP value is displayed in Fig 5. We retained the genes having the shap threshold value > 0.01 for the downstream analysis. The next step was to analyze the genes identified in the STRING database for their protein-protein interaction (PPI) with each other. The constructed PPI networks were subjected to CyberHubba for the extraction of biomarkers based on the MCC presented in Fig 6. Thus, the centrality parameters allow determination of not only the biological significance of the gene but also the gene’s connectivity in the network.

**Fig 5:**
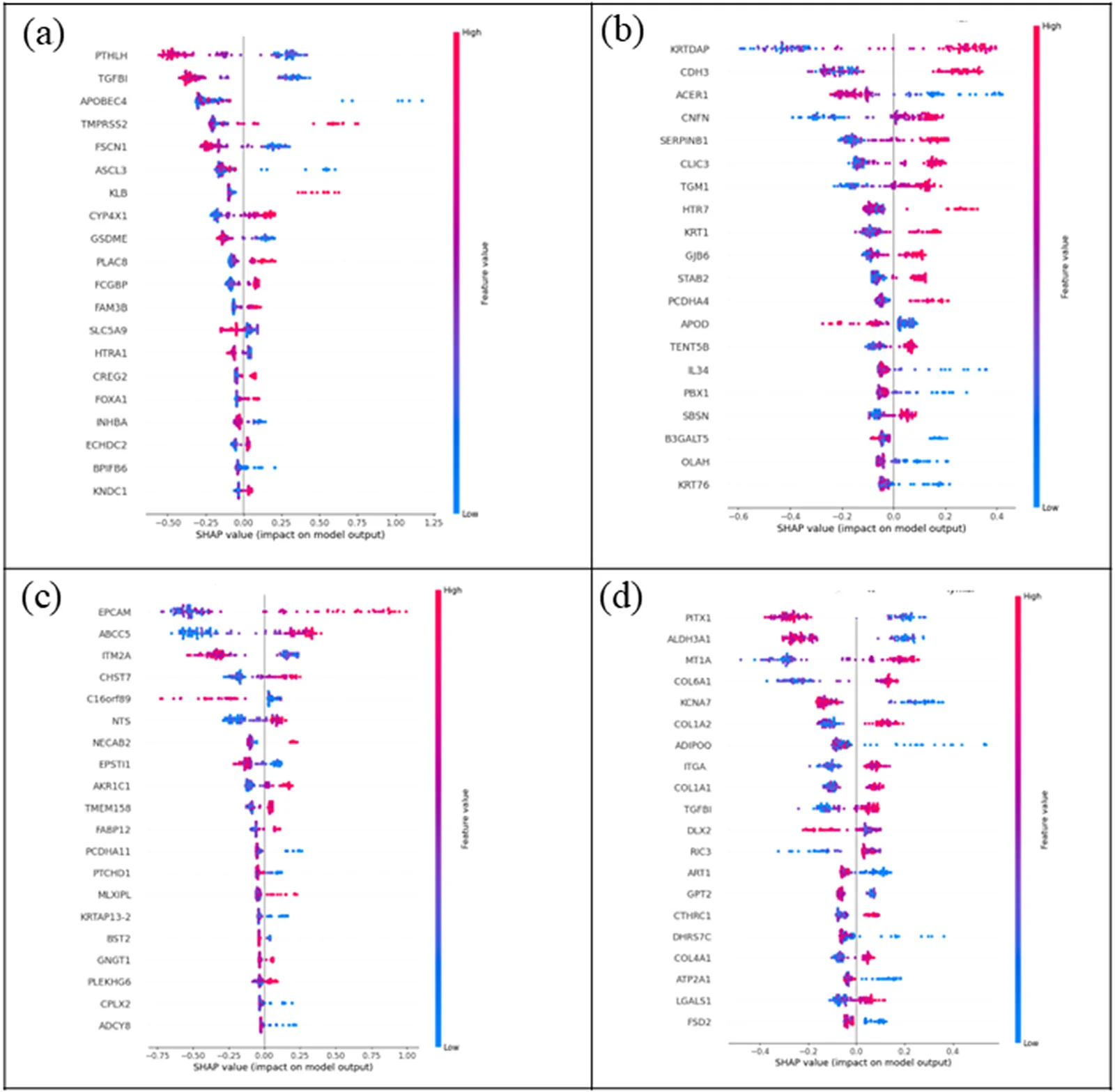
SHAP (SHapley Additive exPlanations) value analysis for subtype-specific genes (a) SHAP summary of the atypical subtype, (b) SHAP summary of the basal subtype, (c) SHAP summary of the classical subtype (d) SHAP summary of the mesenchymal subtype.

**Fig 6:**
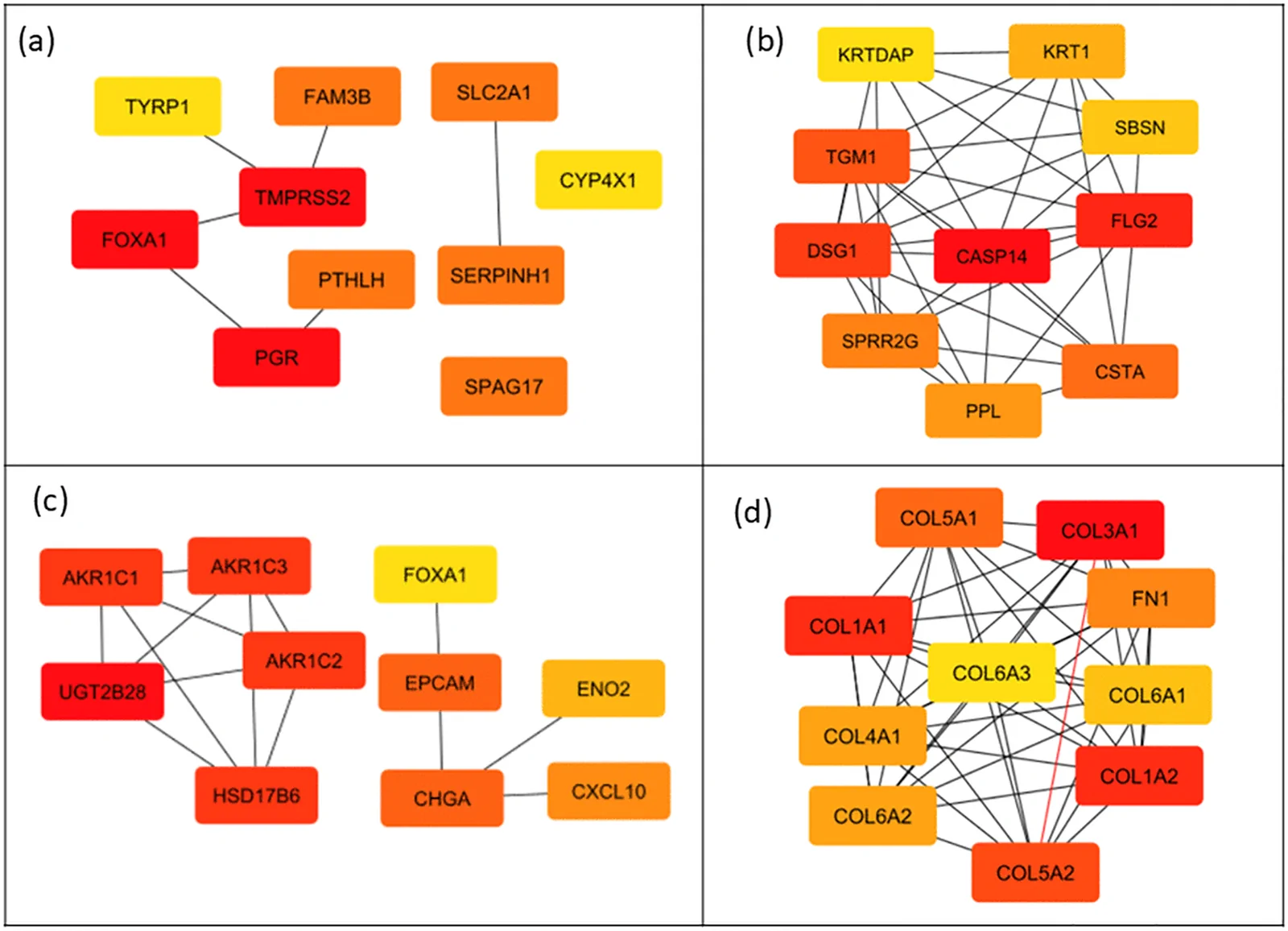
Protein–protein interaction (PPI) networks of subtype-specific hub genes identified using the Maximal Clique Centrality (MCC) algorithm in CytoHubba: (a) Atypical, (b) Classical, (c) Basal, and (d) Mesenchymal molecular subtypes. Nodes represent proteins, and edges represent protein–protein interactions.

A Venn diagram (refer to Fig 7.) of the top-ranked hub genes showed high subclass specificity between the four HNSC molecular subtypes. Ten unique hub genes were identified for both basal and mesenchymal subtypes, and nine were identified for the atypical and classical subtypes. In particular, FOXA1 was the only gene common to both the atypical and classical subtypes, while no common hub genes were identified in the other combinations of subtypes or in all four subtypes. From the joint analysis of CytoHubba across all four subtypes, obtained subtype-specific genes are mentioned in Table 4.

**Table 4:** Subtype-specific genes obtained after the MCC analysis.

Atypical	Basal	Classical	Mesenchymal
TMPRSS2 PGR FAM3B PTHLH SLC2A1 SERPINH1 SPAG17 CYP4X1 TYRP1	CASP14 FLG2 DSG1 TGM1 CSTA SPRR2G PPL KRT1 SBSN KRTDAP	UGT2B28 AKR1C1 AKR1C3 AKR1C2 HSD17B6 EPCAM CHGA CXCL10 ENO2	COL3A1 COL1A1 COL1A2 COL5A2 COL5A1 FN1 COL6A2 COL4A1 COL6A1 COL6A3

**Fig 7:**
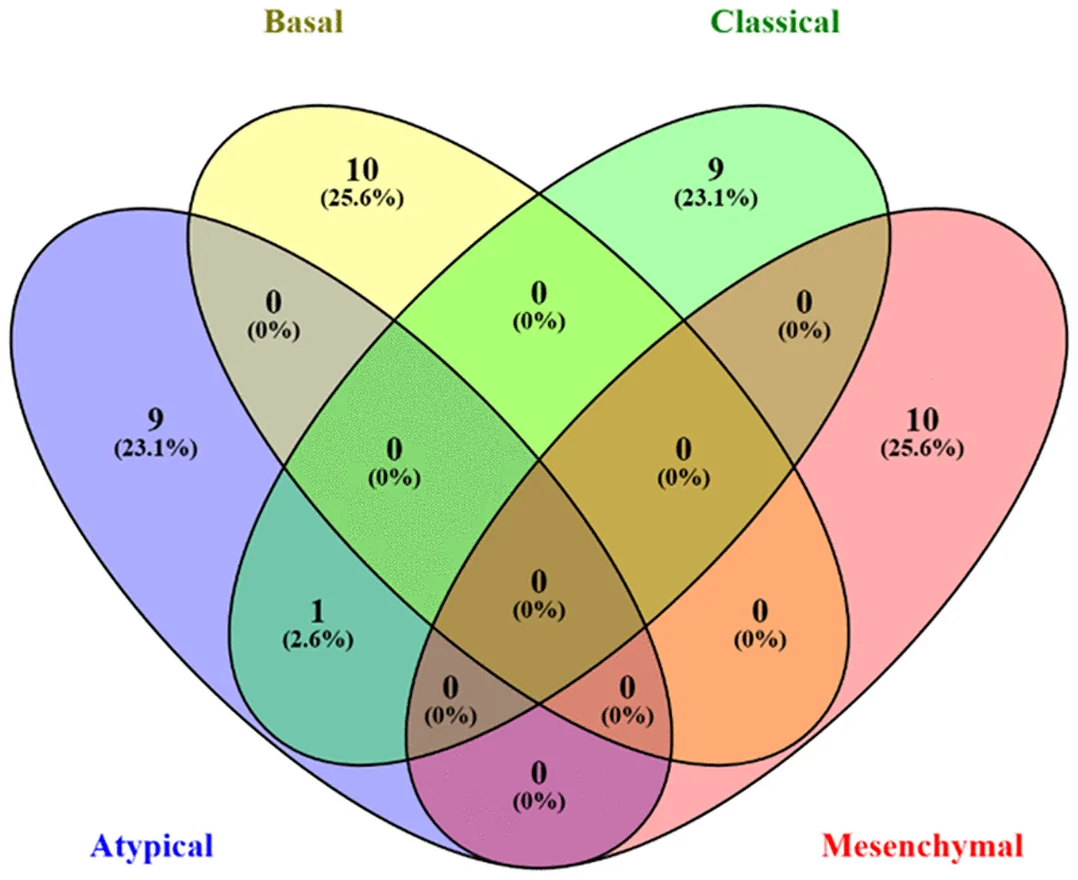
Venn diagram of the subtype-specific biomarkers after the MCC.

### Enrichment analysis using ShinyGO v0.741

The functional enrichment analysis of the biomarkers is given in Fig 8. In the atypical subtype, the biological processes related to chondrocyte development, cartilage development, and glucose metabolism contribute to genes such as PTHLH, SERPINH1, and SLC2A1. Biological processes associated with the basal subtype were highly enriched in functional categories related to epithelial differentiation and keratinization, such as epidermis development, keratinization, keratinocyte differentiation, epithelium development, epidermal cell differentiation, and cornification. These processes were mainly regulated by CASP14, PPL, CSTA, DSG1, FLG2, SPRR2G, KRT1, KRTDAP, and TGM1, which play critical roles in regulating epithelial integrity and terminal differentiation.. Metabolic processes that are most closely associated with ENO2 were also found to be significantly enriched by functional enrichment analysis of the classical subtype, such as glycolytic process, pyruvate metabolic process, carbohydrate catabolic process, nucleotide phosphorylation, purine ribonucleoside diphosphate metabolic process, and phosphopyruvate hydratase complex. Increased cytoplasmic component enrichment (both ENO2 and CHGA) indicates changes in metabolic activity and cellular signaling within the cell. The mesenchymal subtype was characterized by these enriched terms, which were mostly dependent on the recurrent involvement of COL1A1, COL1A2, COL5A1, and FN1, which are genes involved in ECM remodeling. An analysis of the Drug–Gene Interaction database (DGIdb) revealed genes that could serve as druggable biomarkers, thus indicating their importance in a targeted therapeutic approach in head and neck carcinoma. (S1 Table). DGIdb analysis revealed six genes (PGR, TYRP1, PTHLH, SERPINH1, FAM3B, and CYP4X1) that were members of the druggable genome and, therefore, have the potential to be therapeutic targets in the atypical HNSC subtype. These genes were linked to a variety of functional categories, such as nuclear hormone receptors, proteins involved in hormone activity, protease inhibitors, cytochrome P450 enzymes and other enzymes. CASP14, CSTA, KRT1, TGM1, KRTDAP, SBSN, and PPL were identified as genes of potential therapeutic relevance in the basal subtype of HNSC by DGIdb analysis. Of these, CASP14, TGM1, KRTDAP, and SBSN were identified as members of the druggable genome, CSTA as a protease inhibitor, and KRT1 and PPL as kinase-associated targets. These genes were supported by several curated databases, including HingoraniCasas, HopkinsGroom, RussLampel, GuideToPharmacology, Pharos, Human Protein Atlas, Gene Ontology (GO), and dGene. In the classical subtype EPCAM was considered a clinically actionable druggable target, and CXCL10 was annotated as a druggable extracellular protein. These enzymes AKR1C1, AKR1C2, AKR1C3, UGT2B28 and HSD17B6 were identified as druggable enzymes. Furthermore, ENO2 was predicted to be a cell surface-associated protein, and CHGA was predicted to be a part of a druggable genome.For the mesenchymal subtype, COL1A1 is considered a druggable genome, highlighting its strong translational significance for targeted therapy, and COL6A3 was annotated as a protease inhibitor, indicating its potential role in regulating tumor invasion and progression.

**Fig 8:**
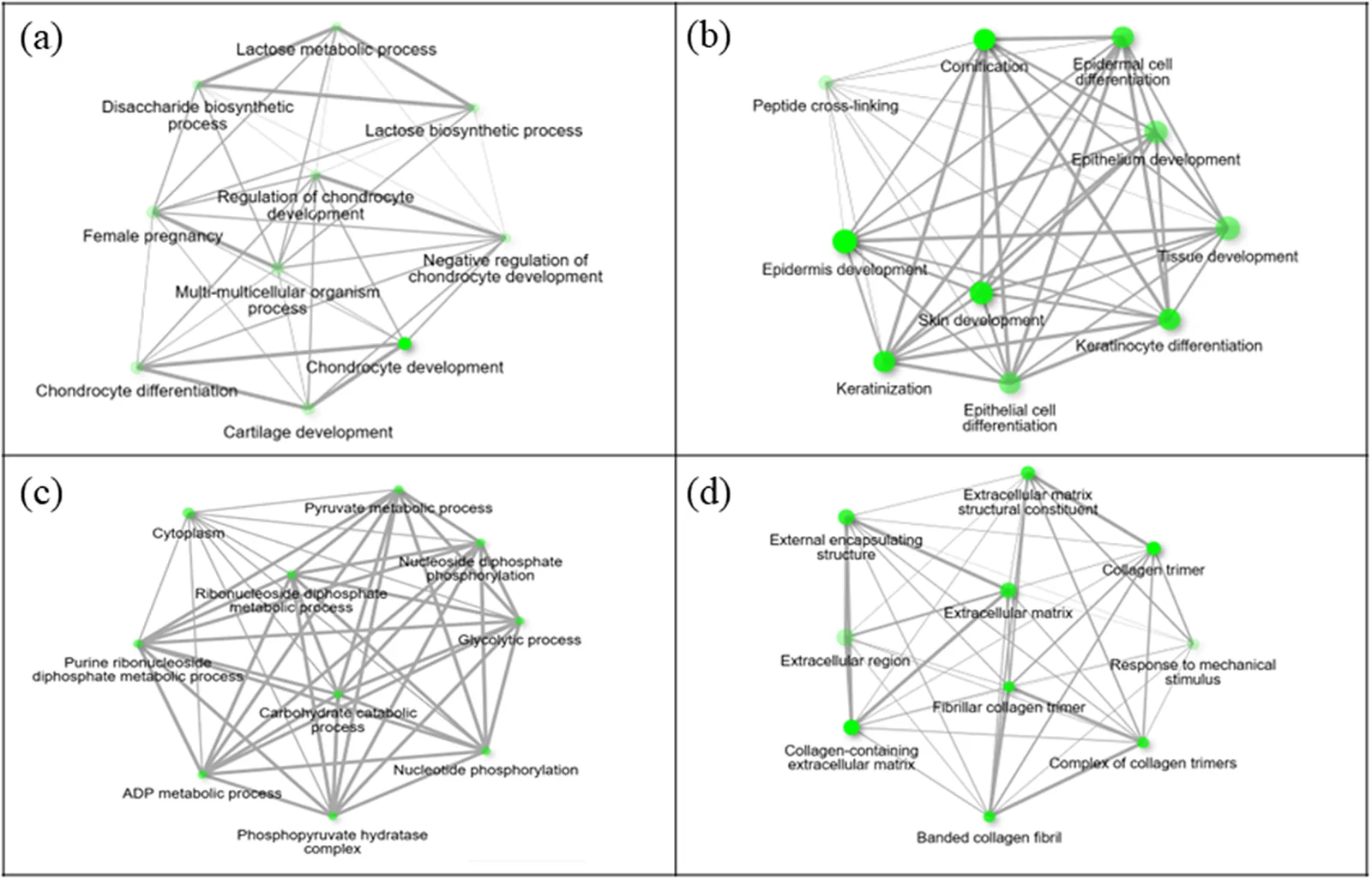
Functional enrichment network of subtype-specific hub genes generated using ShinyGO. Enriched KEGG pathways for the (a) atypical, (b) basal, (c) classical, and (d) mesenchymal molecular subtypes Nodes represent significantly enriched pathways, and edges indicate functional associations between enriched terms.

### GDSC drug sensitivity correlation analysis

The drug resistivity and sensitivity of the subtypes biomarkers shown in the Fig 9. The highest number of significant correlations with several therapeutic agents was observed with the following genes in atypical: SERPINH1, SLC2A1, TMPRSS2, and PTHLH. Most of the positive correlations were with genes involved in kinase inhibitors and targeted compounds, suggesting that high expression levels of these genes are linked to drug resistance, whereas a significant number of negative correlations with clinically relevant EGFR inhibitors (e.g., cetuximab, gefitinib, afatinib) indicate that high expression levels of these genes are linked to drug sensitivity.The classical subtype showed several significant associations with targeted therapies, including EGFR inhibitors (cetuximab, afatinib, gefitinib, erlotinib, and lapatinib), other kinase inhibitors, and epigenetic modulators. The positive and negative correlations between AKR1C1, AKR1C2, AKR1C3, EPCAM, ENO2, CHGA, and CXCL10 suggest a subtype-specific pattern of drug resistance and sensitivity. In the basal subtype, both positive and negative correlations were found with the identified genes in the drug–gene correlation analysis for TH2-102-1. Among the evaluated genes, PPL had the highest association value, indicating a potential resistance-associated relationship, whereas the other five genes (SPRR2G, KRT1, DSG1, CSTA, and CASP14) had negative associations, indicating potential sensitivity-associated relationships.The mesenchymal subtype exhibited the highest number of significant gene–drug associations, indicating high pharmacogenomic heterogeneity. Genes related to the extracellular matrix (ECM) showed primarily positive associations with some chemotherapeutic and targeted agents, such as 5-fluorouracil and docetaxel, and chemotherapeutic agents such as methotrexate, dasatinib, pazopanib, and vorinostat, suggesting that the higher expression of these genes correlates with drug resistance.

**Fig 9:**
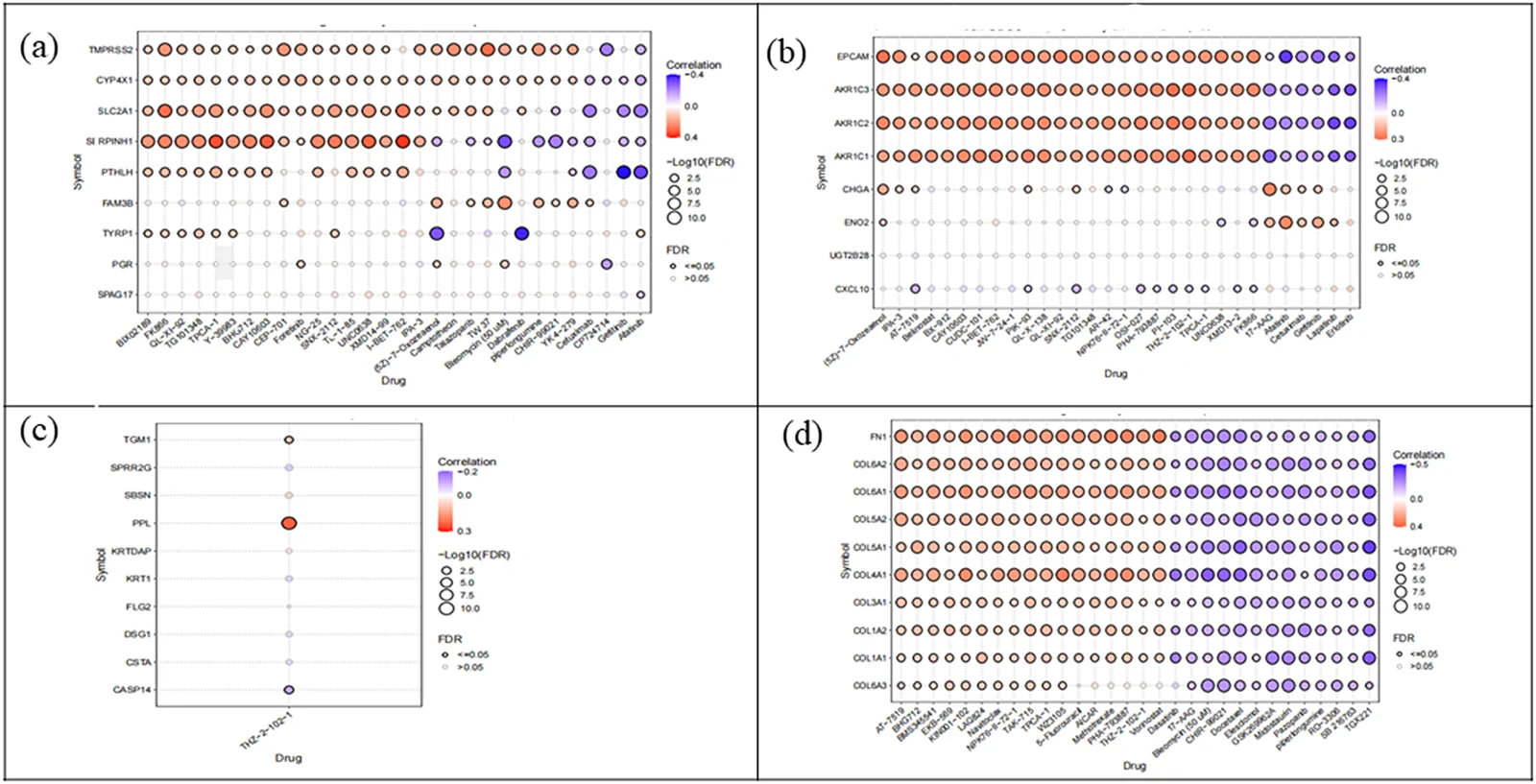
Drug-Resistivity and Sensitivity Analysis of the Subtypes (a) Atypical (b) Classical (c) Basal (d) Mesenchymal.

To assess the prognostic significance, we conducted a survival analysis using the KM Plotter given in S3 Fig - S6 Fig. We considered the parameters such as Hazard Ratio (HR), Confidence Interval (CI) and the P-value (P). In the Atypical subtype, high expression of TMPRSS2 (HR = 0.68, 95% CI: 0.50–0.93, log-rank P = 0.014), FAM3B (HR = 0.69, 95% CI: 0.52–0.91, P = 0.0083), SPAG17 (HR = 0.69, 95% CI: 0.51–0.94, P = 0.017), and CYP4X1 (HR = 0.59, 95% CI: 0.42–0.84, P = 0.0027) was significantly associated with overall survival, indicating a reduced risk of death in patients with elevated expression of these genes. Additionally, SERPINH1 (HR = 1.63, 95% CI: 1.25–2.14, P = 0.00031) and TYRP1 (HR = 1.48, 95% CI: 1.11–1.98, P = 0.0075) were significantly associated with poorer overall survival, indicating that high expression of these genes was linked to increased mortality risk. Although SLC2A1 demonstrated a trend toward adverse prognosis (HR = 1.36, 95% CI: 0.98–1.91, P = 0.067). In the basal subtype, CSTA and SBSN expression levels were significantly associated with overall survival. High expression of CSTA (HR = 0.62, 95% CI: 0.47–0.82, log-rank P = 0.00059) and SBSN (HR = 0.70, 95% CI: 0.51–0.98, P = 0.036) was associated with a reduced risk of death, suggesting that these genes may serve as protective prognostic biomarkers for HNSCC. In the classical subtype, Kaplan–Meier survival analysis revealed variable prognostic importance of subtype-specific genes. CXCL10 was significantly correlated with overall survival (OS) (HR = 0.71, 95% CI: 0.54–0.93, log-rank P = 0.014), with patients with high CXCL10 expression having a lower risk of death than those with low expression levels. AKR1C2 (HR = 1.39, 95% CI: 1.05–1.82, P = 0.019), ENO2 (HR = 1.40, 95% CI: 1.07–1.83, P = 0.014), EPCAM (HR = 1.38, 95% CI: 1.01–1.89, P = 0.043), and AKR1C3 (HR = 1.55, 95% CI: 1.17–2.05, P = 0.002) was significantly associated with poorer overall survival, suggesting that these genes may serve as adverse prognostic indicators in the classical subtype. Kaplan–Meier survival analysis revealed that FN1 was the only gene significantly associated with overall survival in the mesenchymal subtype. High FN1 expression was significantly associated with reduced survival (HR = 1.50, 95% CI: 1.14–1.97, log-rank P = 0.0035), indicating that patients with high FN1 expression had a 50% greater risk of death than those with low FN1 expression. These results indicate that FN1 is the most powerful prognostic gene of the genes in the mesenchymal subtype, while the collagen family genes did not show statistically significant independent association with overall survival in this Kaplan–Meier analysis. The HR value and 95% CI and P value after the KM plotter Survival analysis given in S2 Table.

## Discussions

The four molecular subtypes of HNSCC identified in this study exhibited distinct biological patterns. The classical subtype is characterized by the predominance of EGFR signalling, oxidative phosphorylation, and cell cycle regulation. The basal subtype displays epithelial adhesion and keratin expression. The atypical subtype has lower immune infiltration and different molecular drivers than other subtypes. The mesenchymal subtype is characterized by stromal activation, epithelial–mesenchymal transition (EMT), and immune infiltration [28]. The subtype-specific biomarkers are shown in Table 4. We validated the functional properties using literature. The classical subtypes include biomarkers such as EPCAM, CXCL10, AKR1C1, AKR1C2, AKR1C3, UGT2B28, HSD17B6, ENO2, and CHGA. The head and neck cancer-related evidence for UGT2B28 and CHGA genes is still limited. Aldo-keto reductase AKR1C1, which is involved in steroid metabolism and cellular detoxification, has been associated with treatment resistance and tumor progression in HNSCC. AKR1C1 is a key factor in the development of resistance to cisplatin in HNSCC and the gene enhances survival in tumor cells by activating the STAT1/STAT3 signaling pathway [35]. AKR1C3, a steroid-metabolizing aldo-keto reductase that plays a role in the metabolism of hormones and prostaglandins, is often co-upregulated with AKR1C1 in HPV16-integrated OPSCC. Both AKR1C1 and AKR1C3 have been shown to be upregulated in metabolic dysregulation and tumor progression and significantly linked with poor survival in HPV+ and HPV − HNSCC, indicating their potential role as prognostic and therapeutic markers [36]. Another member of this family of enzymes, AKR1C2, which is also involved in steroid metabolism, has been linked to the clinical features of HNSCC. AKR1C2 expression was significantly associated with AJCC classification and the high expression was linked to improved overall survival in female patients compared to males [37]. HSD17B6 is an enzyme that metabolizes hydroxysteroids and is upregulated in HNSCC [38]. EPCAM is an adhesion molecule present on the surface of epithelial cells and is strongly associated with the epithelial phenotype of HNSCC. EpCAM expression reported in tumors from patients with HNSCC and also the biomarker expression is differentially glycosylated in healthy tissue and tumor cells of the head and neck area [39]. CXCL10 is an interferon-inducible chemokine that recruits CXCR3 positive immune cells and is important in tumor–immune interactions. CXCL10 expression is linked to immune cell infiltration in HNSCC, and CXCL10 cancer-associated fibroblast subsets detected by spatial and single-cell transcriptomic analyzes linked to changes in CD8 + T cell infiltration and dysfunction, suggesting a potential role in regulating the tumor immune microenvironment [40]. A neuron-specific enolase, ENO2, associated with glycolytic metabolism, also associated with metabolic reprogramming in HNSCC. The expression of ENO2 is upregulated in HNSCC and increases the proliferation of tumor cells and glycolysis via ENO2-dependent pathways. Recent studies suggest that ENO2-induced metabolic activity can directly contribute to the polarization of M2 macrophages via histone lactylation and is involved in lymphatic metastasis, suggesting that ENO2 may be important for metabolic reprogramming and tumor–microenvironment interactions [41,42]. Moreover, the metabolic pathway enrichment of nucleotide and ADP metabolism implies that there is likely to be a higher nucleotide turnover to promote ongoing DNA replication and cell proliferation.Further, the metabolic features of this subtype are supported by the presence of ENO2 and CHGA may be involved in cellular signaling and metabolic functions. In the basal subtype, CASP14, FLG2, DSG1, TGM1, CSTA, SPRR2G, PPL, KRT1, SBSN, and KRTDAP were identified as key subtype-associated biomarkers. The CASP14 gene, a member of the caspase family that plays a role in epidermal differentiation, is identified as being extremely responsive to hypoxia in HNSCC. Microarray studies have shown that CASP14 is strong in the hypoxic environment of cell lines derived from HNSCC [43]. FLG2 (filaggrin-2) plays a role in the terminal differentiation of keratinocytes and in maintaining the integrity of the epithelial barrier. FLG2 has been found to be part of keratinization- and epithelial differentiation-associated gene programs in HNSCC, especially in those with a differentiated squamous phenotype [44]. Desmosome-associated cadherin (DSG1) plays a role in the adhesion of epithelial cells and maintenance of the integrity of stratified squamous epithelial tissue. Loss of DSG1 expression has been correlated with shorter disease-specific survival in HNSCC, indicating that impairment of desmosomal adhesion and epithelial integrity may play a role in disease progression [45]. TGM1 (transglutaminase 1) regulates terminal epithelial differentiation and cornified-envelope formation and plays a role in the integrity of stratified squamous epithelium. In HNSCC, changes in TGM1 expression levels have been correlated with tumor and nodal features, and changes in expression between clinical stages and grades indicate a possible association between TGM1 expression and tumor progression and squamous differentiation [46,47]. Cystatin A (CSTA) is a cysteine protease inhibitor that plays a role in epithelial integrity and regulation of cathepsin activity. Lower CSTA expression in HNSCC is correlated with a higher tumor grade, lymph node metastasis, and worse overall survival in OSCCs, suggesting that CSTA may have a protective effect in maintaining epithelial integrity and controlling tumor progression [48]. PPL, also known as periplakin, belongs to the plakin family and is involved in the formation of the cornified envelope and structural integrity of epithelia. PPL has been identified as a hub gene in integrated bioinformatics analyzes of HNSCC, revealing that the gene is downregulated in HNSCC tissues compared with normal tissues [49]. Small proline-rich protein 2G (SPRR2G) plays a role in cornified envelope formation and epithelial keratinization. In HNSCC, SPRR2G is significantly enriched in the transcriptional programs of squamous epithelial and keratinisation, expressed at higher levels in HNSCC than in adjacent normal tissue. The gene is overexpressed in HPV+ HNSCC cell lines, providing additional support for the connection with squamous epithelial differentiation [50]. KRT1 (keratin 1) is a structural keratin associated with terminal squamous epithelial differentiation. KRT1 is linked to recurrence in advanced laryngeal cancers. Additionally, KRT1 knockdown inhibited migration and invasion and promoted apoptosis and drug sensitivity in HNSCC models, indicating that KRT1 may play a role in supporting tumor progression and chemoradioresistance and could be a therapeutic target [51]. SBSN (suprabasin) is an epidermal differentiation-associated protein found in stratified squamous epithelium. Oral squamous cell carcinoma (OSCC): Under hypoxic conditions, SBSN expression is induced and plays a role in cellular proliferation, migration, invasion, and angiogenic activity in OSCC, all of which are more prominent under hypoxia. Furthermore, salivary adenoid cystic carcinoma, another head and neck malignancy, has been demonstrated to exhibit anchorage-dependent and anchorage-independent growth, as well as invasive and metastatic capabilities, and the upregulation of these genes is correlated with hypomethylation in the regulatory region of the gene [52,53]. KRTDAP (keratinocyte differentiation-associated protein) is associated with keratinocyte maturation that is expressed in squamous epithelia. KRTDAP was found to be a potential biomarker for HNSCC and has been shown to be differentially expressed between HPV-negative and HPV-positive HNSCC and healthy tissues [54]. Dysregulation of keratinization and epidermal differentiation pathways is a defining characteristic of basal HNSCC and represents changes in the identity of squamous epithelial cells during tumor progression. Changes in the expression of keratin-associated genes, such as KRT1, and differentiation markers, such as DSG1, PPL, and TGM1, have been linked to the dysfunction of epithelial homeostasis, increased tumor proliferation, and altered cell–cell adhesion. The atypical subtype was associated with key biomarkers, including, TMPRSS2, PGR, FAM3B,PTHLH, SLC2A1, SERPINH1, SPAG17, CYP4X1, TYRP1. TMPRSS2 (transmembrane serine protease 2) is a serine protease expressed on the surface of epithelial cells that participates in the proteolytic processing of cell surface and extracellular substrates. TPMRESS2 is expressed in head and neck tissues and HNSCC, normally associated with epithelial proteolysis and viral entry (e.g., SARS-CoV-2 priming) [55]. Progesterone receptor (PGR) is a nuclear hormone receptor that regulates cell responses to progesterone and plays a significant role in hormone-responsive signaling. A recent study that analyzed primary HNSCC, metastatic lymph nodes, and normal oral tissue found that PGR expression was higher in the metastatic lymph nodes than in the normal tissue, indicating that progesterone signaling may play a role in tumor progression and may be important in the study of HNSCC and its connection to endocrine signaling [56]. FAM3B (family with sequence similarity 3 member B) is a member of the FAM3 cytokine-like family and is involved in cellular signaling and metabolic regulation. In related squamous malignancies, such as esophageal squamous cell carcinoma, higher FAM3B expression has been linked to promoting proliferation, migration, invasion, and epithelial–mesenchymal transition via the AKT–MDM2–p53 signaling pathway [57]. SLC2A1 has been identified in recent HNSCC analyzes of the gene networks of glycolytic and lactate metabolic pathways, mRNA level of SLC2A1 is significantly elevated in HNSCC patients and is associated with shorter overall survival [58]. SPAG17 is a member of the sperm-associated antigen (SPA) family of proteins. SPAG17 was found in tumor tissues, and dendritic cells loaded with SPAG17 antigen were able to generate cytotoxic T cells that kills autologous HNSCC tumor cells in vitro. Moreover, autoantibodies against SP17 have been found in patients’ sera regardless of the tumor location or stage [59]. Serpin family H member 1 (serpin H1, HSP47) is a member of the protein family of endoplasmic reticulum-resident, collagen-specific chaperones involved in collagen folding, maturation, and organization of the extracellular matrix. The study reported that there was significant upregulation of SERPINH1 and correlations with stage, grade, metastases, HPV status, and prognosis [60].

PTHLH (parathyroid hormone-like hormone/PTHrP) is a secreted molecule that functions in tumor cell communication through autocrine and paracrine effects. PTHLH is overexpressed in HNSCC and enhances the cell proliferation. Recent experimental studies on tongue squamous cell carcinoma (SCC) have shown that the removal of tumor-cell-derived PTHLH boosts anti-tumoral immunity, confirming the role in regulating the tumor microenvironment in HNSCC [61]. CYP4X1, also known as cytochrome P450 family 4 subfamily X member 1, is a cytochrome P450 enzyme that metabolizes endogenous physiological substrates and xenobiotic compounds. CYP4X1 has been integrated into prognostic models of HNSCC metastasis, survival prediction, and treatment stratification [62]. TYRP1 (tyrosinase related protein 1) is a melanogenesis associated protein associated with melanin biosynthesis and melanosome biology. A recent transcriptomic analysis of OSCM-derived OSCC revealed that TYRP1 is upregulated in OSCC [63]. SLC2A1 (GLUT1) is especially relevant to HNSCC, as it encodes a glucose transporter that facilitates the increased glycolytic activity of cancer cells. The PTHLH has been linked to tumor progression, invasion, and bone-related metastasis, whereas SERPINH1 is a collagen-specific chaperone that regulates ECM remodeling and has been linked to increased tumor invasiveness and poor prognosis. The Mesenchymal subtype biomarkers are COL3A1, COL1A1, COL1A2, COL5A2, COL5A1, FN1, COL6A2, COL4A1, COL6A1 and COL6A3. The COL3A1 gene encodes the α1 chain of type III collagen, an important structural component of the extracellular matrix (ECM) and a factor that organizes and remodels tissues. COL3A1 was found to be an independent prognostic factor for overall survival (OS) in a TCGA cohort of 502 patients, and this was also confirmed in new clinical samples, suggesting a possible role in HNSCC progression and prognosis [64]. The alpha1 (encoded by COL1A1) and alpha2 (encoded by COL1A2) chains of type I collagen are major structural components of the ECM and important mediators of stromal remodeling. Both have been reported to be upregulated in HNSCC. In addition the expression of epithelial markers suggests the presence of a partial epithelial–mesenchymal transition (pEMT) phenotype that could play a role in tumor invasion and metastasis [65]. The COL5A1 and COL5A2 genes encode components of type V collagen involved in collagen fibril organization and ECM architecture. These genes are associated with alterations in the tumor microenvironment and stromal remodeling in head and neck cancer. Recent studies have shown that increased expression of type V collagen is linked to increased tumor grade and decreased survival in cancer patients and that the expression of type V collagen by CAFs can activate the PI3K/AKT signaling pathway and make the tumor resistant to erlotinib in HNSCC, indicating that remodeling of type V collagen may be linked to both tumor progression and resistance to therapy [66]. Fibronectin (FN1) is a major glycoprotein of the ECM that plays roles in cell adhesion, migration, and tissue remodeling. FN1 is often highly expressed in HNSCC and is correlated with advanced disease and poor prognosis. Functional studies have confirmed its role in the proliferation, migration, and invasion of tumor cells. Moreover, it has been related to cancer-associated fibroblast (CAF)-mediated ECM remodeling and M2 macrophage polarization, which further suggests a role in the induction of a pro-tumorigenic mesenchymal tumor microenvironment (TME) [67]. The three α chains of type VI collagen (COL6A1, COL6A2, and COL6A3) are crucial elements of the ECM and play a role in the organization and stability of the tumor stroma. These three genes are positively correlated with OSCC and highly expressed, suggesting a potential role in tumorigenesis [68]. The COL4A1 gene encodes an important part of type IV collagen, a protein found in basement membranes that is involved in the structure and function of basement membranes and cell-matrix interactions. In HNSCC, COL4A1 is described as a differentially expressed hub gene and has been included in prognostic models, indicating that changes in basement membrane organization and ECM remodeling could play a role in the progression of HNSCC [69]. Collagen remodeling and changes in the extracellular matrix (ECM) are associated with increased epithelial–mesenchymal transition (EMT), cell adhesion, migration, invasion, and metastasis of HNSCC, which are mediated by integrin signaling and increased matrix stiffness.The enrichment of COL1A1, COL1A2, COL5A1 and FN1 in the Mesenchymal subtype underscores the role of ECM remodeling in this molecular phenotype.Survival analysis suggested that SERPINH1, TYRP1, TMPRSS2, FAM3B, SPAG17, and CYP4X1 are favorable-prognosis markers in the atypical subtype. In the classical subtype, survival analysis revealed that AKR1C2, ENO2, CXCL10, EPCAM, and AKR1C3 are the most significant prognostic biomarkers. CSTA and SBSN are the most promising prognostic genes for the basal subtype; FN1 was identified as a prognostic biomarker for the mesenchymal subtype. We conducted an in vitro analysis; however, the validation of the hub genes in a large cohort can strengthen the studies.The comparison of our study with the existing studies is given in Table 5.

**Table 5:** Comparison of our work with existing studies.

Reference	Data Type	ML Approach	Subtypes/Classes Identified	Performance Metrics	Key Findings and Clinical Relevance
[11]	Multi omics	Elastic Net	CS1 and CS2	CS1 exhibited superior survival outcomes	Predicted the prognosis and treatment response of patients
[12]	RNA-seq	5 ML model	CS1 IMU (immune strong) & CS2 KRT (keratinized)	100% accuracy on test set	Developed Tumor subtype classification tool for HPV-associated cancers
[13]	Single-cell RNA-seq	Extreme Gradient Boosting (XGB), Decision Tree (DT), K-Nearest Neighbors (KNN), Extra Trees (ET), Logistic Regression (LR), and Random Forest (RF) algorithms.	HNSCC vs Normal; HPV + vs HPV−	AUROC ~0.91 (HNSCC/Normal), 0.83 (HPV status)	Single-cell biomarkers identified for early detection; deep learning with 100 gene features used. Identified classification of HPV+ and HPV− patients.
[14]	CT Imaging + Clinical Data	MAE-Vision Transformer + Attention (CBAM, BAM) + Fusion	AJCC staging (4 and 5 stages)	80.4% accuracy and 0.83 AUC	Effective multimodal fusion for stage classification; attention mechanisms adapt to data sparsity
**Our Model**	Transcriptomic subtype Data (TCGA +GEO)	Employed 9 ML models for subtype classification	Mesenchymal, classical, basal and atypical	Out of 9 models, XGBoost reached an accuracy of 95%	Developed a subtype-based ML classification model using XAI and established a gene signature for each subtype

### Limitations and future perspectives

The study relied on publicly available datasets such as TCGA and GEO; however, the availability of larger groups with well-defined molecular subtype annotations of HNSCC is one of the significant limitations. Future studies may include larger multi-center cohorts to increase the generalizability and robustness of the proposed framework. Furthermore, the present study can be expanded to population-specific factors, such as race/ethnicity and geographic location to understand its role in the disease and possible newer genetic findings. The proposed machine learning framework included feature selection, class balancing, and cross-validation to make the model more robust; however, the possibility of residual overfitting in a high dimensional transcriptomic analysis is always present. Moreover, the identified subtype-specific biomarkers showed therapeutic significance and were confirmed by druggability assessment via DGIdb and functional enrichment analysis. The hypothetical analysis efficiently identifies candidate markers and therapeutic targets in a time-efficient manner. These computational data must be interpreted with caution as evidence of therapeutic efficacy and need to be tested experimentally to establish their biological and therapeutic significance. The multivariable survival analyzes that include detailed clinical covariates would provide additional prognostic based validation of the clinical relevance of these biomarkers. Subsequent studies involving multi-omics analysis, single cell and spatial transcriptomics, proteomics, and longitudinal clinical information will further help to refine the grouping of subtypes, identification of biomarkers, and precision therapeutic targets.

## Conclusion

This study developed a systems-level framework for HNSCC subtype-specific biomarker discovery using XAI. Molecular subtype classification using machine learning has revealed unique molecular characteristics related to the known transcriptional phenotypes of HNSCC, including classical, basal, mesenchymal, and atypical phenotypes, indicating various biological characteristics. The XGBoost classifier provided the better classification accuracy of 95%, which underlines the models robustness in dealing with high-dimensional omics data and interpretability in feature prioritization. These putative gene biomarkers suggest the presence of specific molecular interaction networks for each subtype of HNSCC. In addition, the expression of FOXA1 indicates the shared role of being a potential common regulator between both atypical and classical subtypes. This information may be more helpful for exploring underlying molecular mechanisms among subtypes regulation. These findings highlight the molecular heterogeneity of HNSCC and the need for subsequent functional characterization for therapeutic prioritization. The integrative approaches can help translate a molecular signature identified using computational analysis into a clinically relevant biomarker that can be used for the personalized management of HNSCC.

## Supporting information

S1 Fig Principal component analysis (PCA) of the combined HNSCC datasets before and after batch-effect correction. (a) Before correction. (b) After correction.
(TIF)

S2 Fig Confusion matrices of the machine-learning models used for HNSCC subtype classification. (TIF)

S3 Fig Kaplan–Meier survival analysis of HNSCC atypical molecular subtype using KMplotter. (TIF)

S4 Fig Kaplan–Meier survival analysis of HNSCC basal molecular subtype using KMplotter. (TIF)

S5 Fig Kaplan–Meier survival analysis of HNSCC classical molecular subtype using KMplotter. (TIF)

S6 Fig Kaplan–Meier survival analysis of HNSCC mesenchymal molecular subtype using KMplotter. (TIF)

S1 Table Potential druggable biomarkers identified using the Drug–Gene Interaction Database (DGIdb). (PDF)

S2 Table Kaplan–Meier Plotter survival analysis reporting hazard ratio (HR), 95% confidence interval (95% CI), and P value. (PDF)

S1 Data Gene expression data from the TCGA and GEO datasets. (CSV)
