# Supplementary material for: Explainable computational framework for subtype classification and biomarker identification in head and neck squamous cell carcinoma using machine learning
Source: PLoS One. 2026 Nov 4;21(11):e0360612. doi: 10.1371/journal.pone.0360612 (PMC13734489; doi:10.1371/journal.pone.0360612)
Supplement: Supplementary file 1 [file pone.0360612.s007.pdf]

S1 Table 1. Potential druggable biomarkers identified using the Drug–Gene Interaction Database (DGIdb)

Atypical

**Gene**	**DGIdb Category**	**Source Database(s)**
PGR	Druggable Genome<br><br>Nuclear Hormone Receptor	HingoraniCasas, HopkinsGroom, RussLampel<br><br>Gene Ontology (GO), GuideToPharmacology, HopkinsGroom, dGene
TYRP1	Druggable Genome<br><br>Enzyme	HingoraniCasas, RussLampel<br><br>Pharos
PTHLH	Druggable Genome<br><br>Hormone Activity	HingoraniCasas<br><br>Gene Ontology (GO)
SERPINH1	Druggable Genome<br><br>Protease Inhibitor	HopkinsGroom, RussLampel<br><br>HopkinsGroom, dGene
	Druggable Genome<br><br>External Side of Plasma Membrane<br><br>Transporter	HingoraniCasas, RussLampel<br><br>Gene Ontology (GO)<br><br>GuideToPharmacology, Human Protein Atlas, Pharos
FAM3B	Druggable Genome	HingoraniCasas

CYP4X1	Cytochrome P450<br>Druggable Genome<br>Enzyme	HopkinsGroom<br>HingoraniCasas, HopkinsGroom, RussLampel<br>GuideToPharmacology, Pharos

Basal

**Gene**	**DGIdb Category**	**Source Database(s)**
CASP14	DRUGGABLE GENOME<br>ENZYME<br>PROTEASE	HopkinsGroom RussLampel<br>GuideToPharmacology HumanProteinAtlas Pharos<br>GO HopkinsGroom dGene
CSTA	PROTEASE INHIBITOR	dGene
KRT1	KINASE	Pharos
TGM1	DRUGGABLE GENOME<br>ENZYME	HingoraniCasas HopkinsGroom RussLampel<br>HumanProteinAtlas
KRTDAP	DRUGGABLE GENOME	HingoraniCasas
SBSN	DRUGGABLE GENOME	HingoraniCasas
PPL	KINASE	Pharos

Classical

Gene	**DGIdb Category**	**Source Database(s)**
EPCAM	CELL SURFACE<br>CLINICALLY ACTIONABLE<br>DRUGGABLE GENOME	GO<br>MskImpact Tempus<br>HingoraniCasas
CXCL10	DRUGGABLE GENOME	HingoraniCasas<br>GO

	EXTERNAL SIDE OF PLASMA MEMBRANE	
AKR1C3	DRUGGABLE GENOME<br><br>ENZYME	HingoraniCasas HopkinsGroom RussLampel<br><br>GuideToPharmacology Pharos
AKR1C1,AKR1C2	DRUGGABLE GENOME<br><br>ENZYME	HingoraniCasas HopkinsGroom RussLampel<br><br>Pharos
ENO2	CELL SURFACE	GO
CHGA	DRUGGABLE GENOME	HingoraniCasas
UGT2B28	DRUGGABLE GENOME<br><br>ENZYME	HingoraniCasas<br><br>Pharos
HSD17B6	DRUGGABLE GENOME<br><br>SHORT CHAIN DEHYDROGENASE REDUCTASE	HopkinsGroom RussLampel<br><br>HopkinsGroom

Mesenchymal

Gene	DGIdb Category	Source Database(s)
FN1	DRUGGABLE GENOME	HingoraniCasas
COL1A2	DRUGGABLE GENOME	HingoraniCasas
COL1A1	CLINICALLY ACTIONABLE<br><br>DRUGGABLE GENOME	CarisMolecularIntelligence FoundationOneGenes<br><br>HingoraniCasas
COL3A1, COL4A1 COL5A1, COL5A2	DRUGGABLE GENOME	HingoraniCasas
COL6A1 , COL6A2	DRUGGABLE GENOME	HingoraniCasas RussLampel
COL6A3	DRUGGABLE GENOME	HingoraniCasasRussLampel

	PROTEASE INHIBITOR	dGene
