# Supplementary material for: Explainable computational framework for subtype classification and biomarker identification in head and neck squamous cell carcinoma using machine learning
Source: PLoS One. 2026 Nov 4;21(11):e0360612. doi: 10.1371/journal.pone.0360612 (PMC13734489; doi:10.1371/journal.pone.0360612)
Supplement: Supplementary file 2 [file pone.0360612.s008.pdf]

S2 Table Kaplan–Meier Plotter survival analysis reporting hazard ratio (HR), 95% confidence interval (95% CI), and *P* value

Atypical subtype genes	Hazard Ratio (HR)	95% CI	P value
TMPRSS2	0.68	0.50–0.93	0.014
PGR	0.88	0.67–1.14	0.33
FAM3B	0.69	0.52–0.91	0.0083
PTHLH	1.18	0.86–1.61	0.31
SLC2A1	1.36	0.98–1.91	0.067
SERPINH1	1.63	1.25–2.14	0.00031
SPAG17	0.69	0.51–0.94	0.017
CYP4X1	0.59	0.42–0.84	0.0027
TYRP1	1.48	1.11–1.98	0.0075

Basal subtype genes	Hazard Ratio (HR)	95% CI	P value
CASP14	1.13	0.86–1.47	0.390
SPRR2G	0.87	0.67–1.14	0.320
PPL	0.82	0.63–1.07	0.140
TGM1	0.80	0.60–1.07	0.130
SBSN	0.70	0.51–0.98	0.036
KRTDAP	0.74	0.53–1.02	0.068

KRT1	0.82	0.62–1.09	0.170
FLG2	0.89	0.67–1.19	0.440
DSG1	0.79	0.57–1.08	0.140
CSTA	0.62	0.47–0.82	0.00059

Classical subtype genes	Hazard Ratio (HR)	95% CI	P value
UGT2B28	0.72	0.47–1.09	0.120
CXCL10	0.71	0.54–0.93	0.014
HSD17B6	0.80	0.61–1.04	0.098
AKR1C2	1.39	1.05–1.82	0.019
ENO2	1.40	1.07–1.83	0.014
CHGA	1.16	0.86–1.57	0.340
EPCAM	1.38	1.01–1.89	0.043
AKR1C1	1.30	0.98–1.72	0.071
AKR1C3	1.55	1.17–2.05	0.002

Mesenchymal subtype genes	Hazard Ratio (HR)	95% CI	P value
COL3A1	1.18	0.88–1.59	0.25
COL1A1	1.18	0.88–1.58	0.27
COL1A2	0.87	0.66–1.15	0.33
COL5A2	1.24	0.93–1.64	0.14

COL5A1	1.24	0.95–1.62	0.11
FN1	1.50	1.14–1.97	0.0035
COL6A2	1.14	0.86–1.49	0.36
COL4A1	0.87	0.67–1.14	0.32
COL6A1	1.11	0.84–1.46	0.46
COL6A3	0.86	0.65–1.14	0.29
